# A Case of Emphysema

**Published:** 1787

**Authors:** John Darby

**Affiliations:** Surgeon at Diss, in Norfolk


VI. *A Caſe of Emphyſema. Communicated in a Letter to Dr.* Simmons *by Mr.* John Darby, *jun. Surgeon at Diſs, in Norfolk.*

ROBERT Roper, an induſtrious farmer, of the pariſh of Wortham, in the county of Suffolk, aged ſeventy-five years, had the misfortune, in aſſiſting to unload beans the latter end of September laſt, to fall from a waggon, and ſtrike his right ſide againſt a ladder. As the accident happened late in the evening, and the patient was able to reach his houſe without experiencing much pain, he ſuppoſed that the injury received was of no material conſequence, and that its effects would be eaſily obviated by reſting a few hours in bed. Early the next morning, however, he was ſeized with a conſiderable degree of anxiety about the breaſt, and his reſpiration became difficult and laborious. I was deſired, therefore, to viſit him, and, upon examining the part which had ſuſtained the injury, was ſurpriſed to find a tumor of of an enormous fize fituated on the pectoral mufcle. This tumor, at firft fight, I conjectured to be occafioned by extravafated blood, but, on preffing it, found it produced a crackling noife, which, with the rapid progrefs of the fwelling, together with the flatulent feel of the part, induced me to fuppofe that it was emphyfematous, and occafioned by a fractured rib, which had pierced the pleura, and the fpiculæ of which had wounded the lungs. Upon a more minute examination, I found all the cellular membrane diftended with air on the neck and cheek of that fide on which he had received the injury.

To prevent inflammation, I bled him, and was aftonifhed to find how faft every part of his body partook of the emphyfema during the time he underwent the operation. Notwithftanding I had never feen an emphyfema, except in gangrenous parts, I recollected to have perufed a cafe attended with fimilar circumftances, publifhed by Dr. Hunter in the fecond volume of the Medical Obfervations and Inquiries; and I refolved to purfue the method of treatment there recommended. I immediately, therefore, with the fhoulder of my lancet, made an incifion, two inches long, through the fkin and cellular cellular membrane, over the fractured rib. The air rushed out immediately, and continued to be heard to do so for some considerable time, just as it does when forced through the mouth of a pair of bellows. I dipped my hand in some oily embrocation, and continued to stroke the surrounding parts with it for some time, by which means the tumor was considerably diminished; I then dressed the wound superficially, and applied a napkin, dipped in a cold mixture of brandy and vinegar, tightly round the breast, and over that the scapulary bandage.

The patient complained of a violent pain in his side, and coughed frequently, but did not raise any blood. To alleviate these symptoms, I directed pectoral medicines, with small doses of tinct. thebaic, to be frequently given, and a liberal use of diluting liquors.

Upon visiting him the next morning, I was informed he had passed a good night, but found that more air had escaped from the lungs into the cellular membrane, so as to distend the opposite side; and his eyelids were so much swelled, that he had not been able to see for several hours. The penis and scrotum likewise partook of the distention, which was now become general, and exhibited an appearance ſimilar to that of a ſtuffed body. Under theſe circumſtances, I did not heſitate to make more inciſions, and punctured the ſkin of the oppoſite breaſt, of the ſcrotum, and penis; and afterwards of the eyelids, by which means he was immediately enabled to ſee.

I deſired the nurſe to remove the dreſſings in the evening, and to preſs out the air as much as poſſible by rubbing the ſurrounding parts, and it was wonderful the quantity of air that was conſtantly emitted.

The following day I was pleaſed at finding a conſiderable diminution of every part; but as the patient ſtill breathed with difficulty, I was induced to have recourſe again to venæſection. This had a good effect, and at the end of ten days I had the pleaſure of finding him quite well, and he now enjoys as good health as he has experienced for ſeveral years.

*Diſs,*

Nov. 18th, 1787.

